# About the existence of common determinants of gene expression in the porcine liver and skeletal muscle

**DOI:** 10.1186/s12864-019-5889-5

**Published:** 2019-06-24

**Authors:** Rayner González-Prendes, Emilio Mármol-Sánchez, Raquel Quintanilla, Anna Castelló, Ali Zidi, Yuliaxis Ramayo-Caldas, Tainã Figueiredo Cardoso, Arianna Manunza, Ángela Cánovas, Marcel Amills

**Affiliations:** 1 Department of Animal Genetics, Centre for Research in Agricultural Genomics (CRAG), CSIC-IRTA-UAB-UB, Universitat Autònoma de Barcelona, Bellaterra, 08193 Barcelona, Spain; 2 Animal Breeding and Genetics Program, Institute for Research and Technology in Food and Agriculture (IRTA), Torre Marimon, 08140 Caldes de Montbui, Spain; 3 Departament de Ciència Animal i dels Aliments, Facultat de Veterinària, Universitat Autònoma de Barcelona, 08193 Bellaterra, Spain; 4 CAPES Foundation, Ministry of Education of Brazil, Brasilia D. F, Zip Code 70.040-020 Brazil; 5 Centre for Genetic Improvement of Livestock, Department of Animal Biosciences, University of Guelph, 50 Stone Road East, Guelph, Ontario N1G 2W1 Canada; 6 Departament de Producció Animal-Agrotecnio Center, Universitat de Lleida, 191 Rovira Roure, 25198 Lleida, Spain

## Abstract

**Background:**

The comparison of expression QTL (eQTL) maps obtained in different tissues is an essential step to understand how gene expression is genetically regulated in a context-dependent manner. In the current work, we have compared the transcriptomic and eQTL profiles of two porcine tissues (skeletal muscle and liver) which typically show highly divergent expression profiles, in 103 Duroc pigs genotyped with the Porcine SNP60 BeadChip (Illumina) and with available microarray-based measurements of hepatic and muscle mRNA levels. Since structural variation could have effects on gene expression, we have also investigated the co-localization of *cis-*eQTLs with copy number variant regions (CNVR) segregating in this Duroc population.

**Results:**

The analysis of differential expresssion revealed the existence of 1204 and 1490 probes that were overexpressed and underexpressed in the *gluteus medius* muscle when compared to liver, respectively (|fold-change| > 1.5, *q-value* < 0.05). By performing genome scans in 103 Duroc pigs with available expression and genotypic data, we identified 76 and 28 genome-wide significant *cis-*eQTLs regulating gene expression in the *gluteus medius* muscle and liver, respectively. Twelve of these *cis*-eQTLs were shared by both tissues (i.e. 42.8% of the *cis-*eQTLs identified in the liver were replicated in the *gluteus medius* muscle). These results are consistent with previous studies performed in humans, where 50% of eQTLs were shared across tissues. Moreover, we have identified 41 CNVRs in a set of 350 pigs from the same Duroc population, which had been genotyped with the Porcine SNP60 BeadChip by using the PennCNV and GADA softwares, but only a small proportion of these CNVRs co-localized with the *cis*-eQTL signals.

**Conclusion:**

Despite the fact that there are considerable differences in the gene expression patterns of the porcine liver and skeletal muscle, we have identified a substantial proportion of common *cis-*eQTLs regulating gene expression in both tissues. Several of these *cis*-eQTLs influence the mRNA levels of genes with important roles in meat (*CTSF*) and carcass quality (*TAPT1*), lipid metabolism (*TMEM97*) and obesity (*MARC2*), thus evidencing the practical importance of dissecting the genetic mechanisms involved in their expression.

**Electronic supplementary material:**

The online version of this article (10.1186/s12864-019-5889-5) contains supplementary material, which is available to authorized users.

## Background

The performance of GWAS in humans has revealed that most of the regions that display significant associations with complex traits are not exonic, meaning that regulatory polymorphisms might have important effects on phenotypic variation [1]. This realization has prompted the mapping of expression QTL (eQTL), i.e. single nucleotide polymorphisms (SNP), indels or copy number variants (CNV) that explain part of the variance of gene expression phenotypes [1]. Such studies have revealed that the majority of eQTL exert their effects in *cis*- (i.e. on neighboring genes) [2]. There are also evidences that CNV-tagging SNPs are enriched in *cis*-eQTLs and that they often modulate multiple expression traits [3]. By examining the patterns of expression of 22,286 genes in 9 human tissues, the GTEx Consortium has shown that approximately 50% of eQTL are shared by the nine tissues and that most of them display consistent effects across tissues [4].

In pigs, hundreds of eQTLs with effects on muscle [5–9], liver [10, 11] and backfat [12] gene expression have been mapped. Often, these pig eQTL studies have targeted subsets of genes either mapping to QTL [12, 13] or displaying significant expression-phenotype correlations [5, 6, 10, 11]. The broad majority of porcine eQTL studies have targeted single anatomic locations and, in consequence, they do not provide clues about the differential genetic regulation of distinct tissues and organs. Moreover, these genome scans have explored the association of gene expression with the allelic variation of SNPs or microsatellites, neglecting the potential effect of CNVs on expression phenotypes. The goals of the current work were to compare the gene expression patterns and *cis-*eQTL landscapes of the pig *gluteus medius* (GM) skeletal muscle and liver, two tissues with highly differentiated patterns of expression [13], as well as to investigate the co-localization of *cis-*eQTLs and CNVRs.

## Results

### Differential expression analysis

**Fig. 1:**
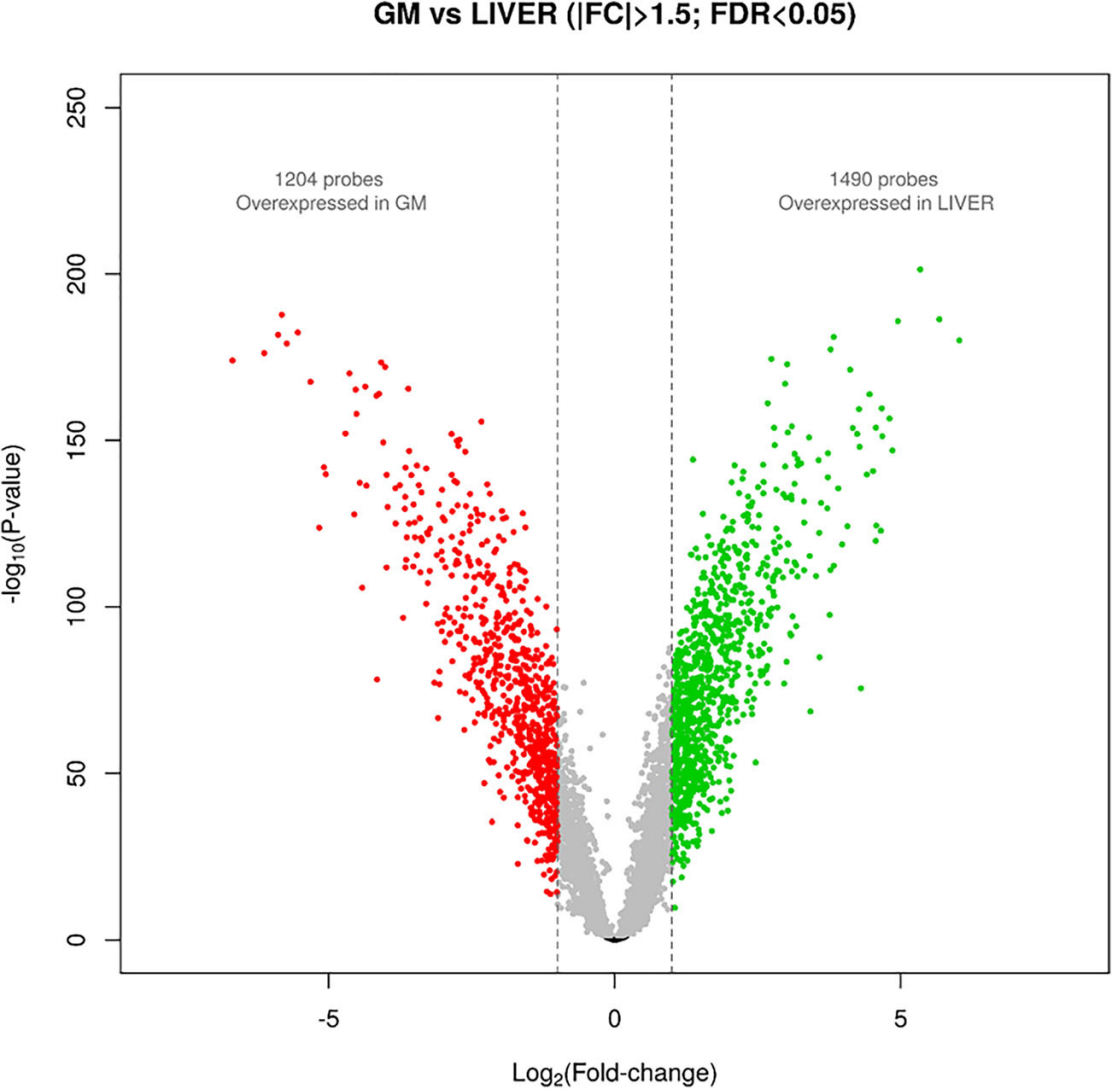
Volcano plot depicting porcine genes that are differentially expressed in the porcine *gluteus medius* muscle and the liver

A total of 1204 probes were found to be overexpressed in the GM muscle (|FC| > 1.5; *q-value* < 0.05), whereas 1490 showed overexpression in the liver (Fig. 1 and Additional file 1). The list of genes showing the highest levels of differential expression between muscle and liver, included the bridging integrator 1 (*BIN1*, log_2_FC = 5.34, *q-value* = 2.21E-198), the sorbitol dehydrogenase (*SORD*, log_2_FC = − 5.82, *q-value* = 3.47E-185), the protein phosphatase 1 regulatory inhibitor subunit 1A (*PPP1R1A*, log_2_FC = 5.67, *q-value* = 5.62E-184), the zinc finger protein 106 (*ZNF106*, log_2_FC = 4.94, *q-value* = 1.60E-183), the hydroxysteroid 17-beta dehydrogenase 13 (*HSD17B13*, log_2_FC = − 5.54, *q-value* = 3.04E-180) and apolipoprotein A1 (*APOA1*, log_2_FC = − 5.88, *q-value* = 1.25E-179). Noteworthy, the log_2_FC values are very high and the *q-value*s highly significant, thereby evidencing that the expression profiles of the porcine liver and the skeletal muscle are strongly divergent.

### Detection of expression QTL in the porcine skeletal muscle and liver

**Fig. 2:**
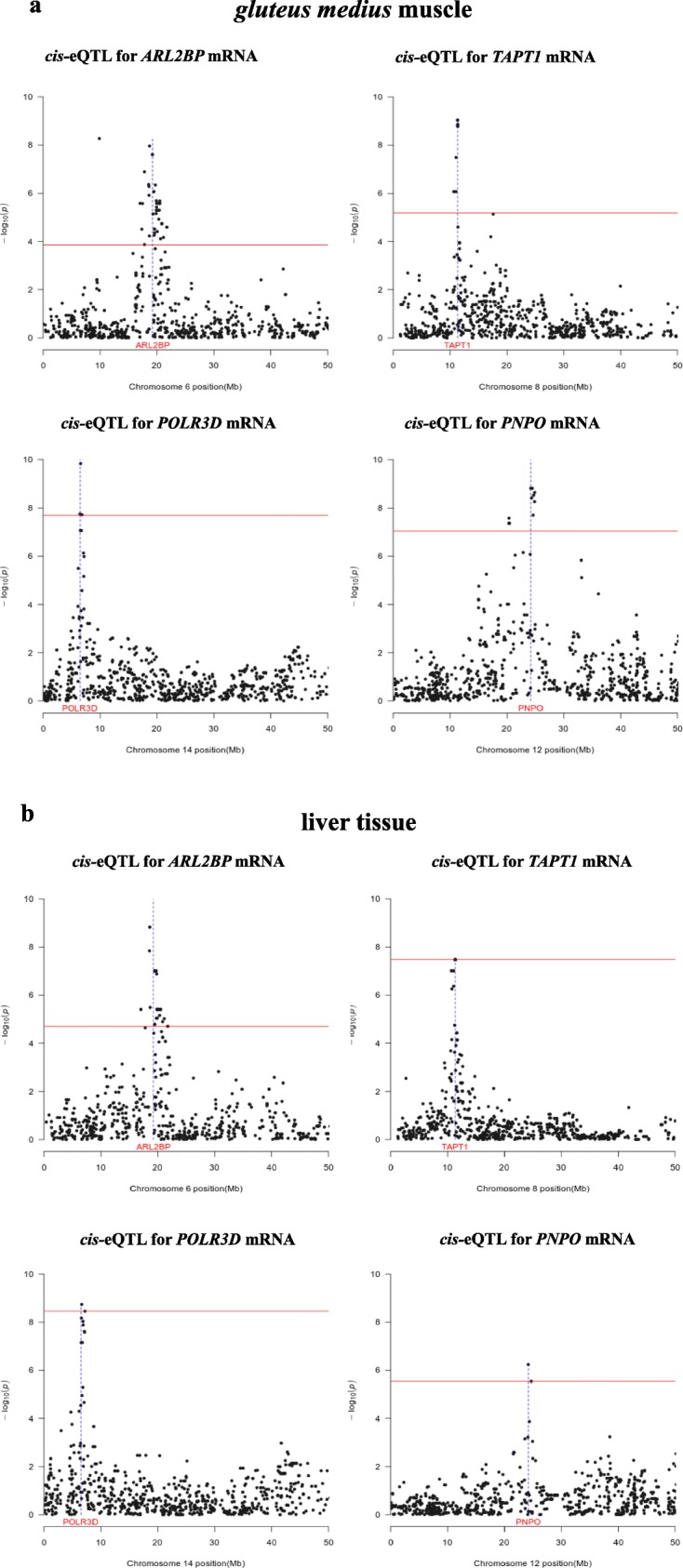
Examples of genes that are regulated by the same *cis-*eQTL in the *gluteus medius* muscle (**a**) and liver (**b**). In the Manhattan plots, the horizontal line indicates the threshold of significance after correction for multiple testing, whilst the vertical line depicts the genomic location of the four genes (*ARL2BP, TAPT1, POLR3D* and *PNPO*) under consideration

**Fig. 3:**
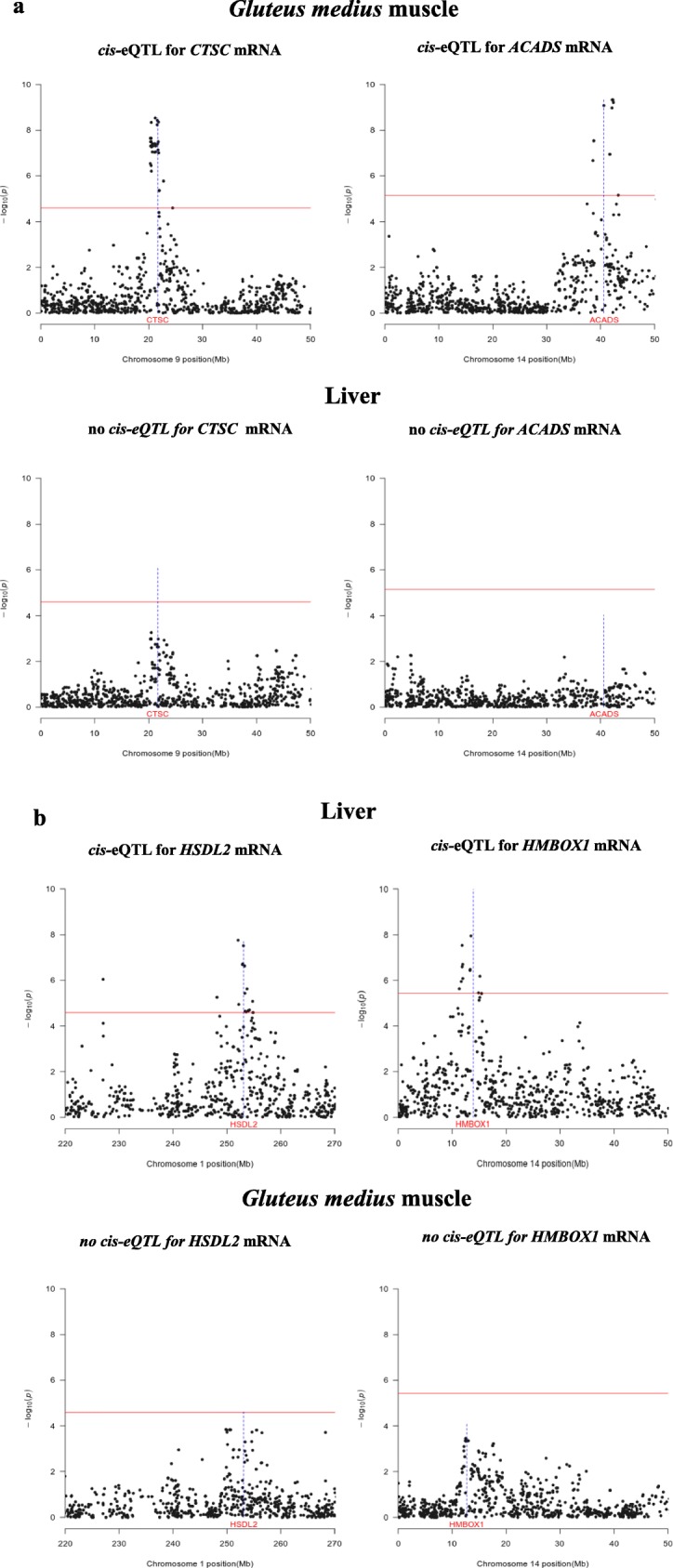
Examples of *cis-*eQTLs that are found in the muscle but not in the liver (**a**) and vice versa (**b**). In the Manhattan plots, the horizontal line indicates the threshold of significance after correction for multiple testing, whilst the vertical line depicts the genomic location of the *CTSC*, *ACADS*, *HSDL2* and *HMBOX1* genes

**Fig. 4:**
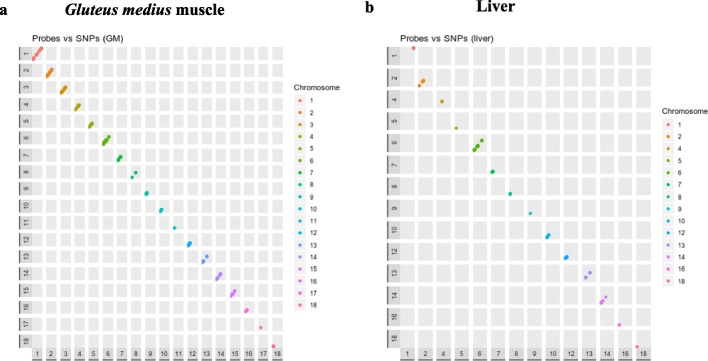
Genomic position of *cis-*regulated probes vs genomic positions of SNPs significantly associated with the expression of such probes in the muscle (**a**) and in the liver (**b**). Diagonal dots represents *cis*-eQTLs (dots from different chromosomes have different colors)

A total of 76 and 28 genome-wide significant *cis*-eQTLs were identified in the GM muscle and liver*,* respectively (Tables 1 and 2). Several genes that are *cis*-regulated in the muscle participate in lipid or carbohydrate metabolism, e.g. HIV-1 Tat Interactive Protein 2 (*HTATIP2*) [14], exocyst complex component 7 (*EXOC7*) [15] acyl-CoA dehydrogenase short chain (*ACADS*) [16], insulin degrading enzyme (*IDE*) [17], solute carrier family 38 member 9 (*SLC38A9*) [18], and family with sequence similarity 3 member C (*FAM3C*) [19]. With regard to the liver, hydroxysteroid dehydrogenase like 2 (*HSDL2*) [20] and lipase A (*LIPA*) [21] genes have also important roles in lipid metabolism. The level of overlap between the muscle and the liver *cis*-eQTL data sets was considerable, with 12 *cis-*eQTLs shared by both tissues (42.8% of the *cis-*eQTLs detected in the liver were also detected in the GM muscle). Four examples of shared *cis-*eQTLs are shown in Fig. 2. There was a strong consistency in the direction of the effects of the shared *cis*-eQTLs in both tissues. For instance, the *cis-*eQTL regulating *ARL2BP* and *TMEM97* showed positive allelic effects in both tissues, whilst consistent negative allelic effects were estimated for the *cis*-eQTL modulating the hepatic and muscular expression of *BSDC1*, *POLR3D* and *TAP1* (Tables 1 and 2). We also detected a number of *cis-*eQTLs that had effects on gene expression either in the muscle or in the liver (Figs. 3 and 4). RNA-Seq data from the GM muscle from 52 of the 103 pigs with GM muscle microarray was also available. Comparison of the genotypic means obtained with both platforms and corresponding to genes that are *cis*-regulated in the GM muscle provided highly consistent results, i.e. the direction of the difference between the two homozygous genotypes was concordant in 82% of the comparisons and the same ordering of genotypes (from highest to lowest expression) was obtained in 70% of the comparisons (Additional file 2). However, both methods did not always yield significant results (about 50% of coincidence with regard to statistical significance). The most probable reason for this is that the sample size of the RNA-Seq data set is half the size when compared to the microarray data set, which leads to larger standard deviations for the RNA-Seq datasets. This would consequently reduce the power to detect significant differences between the genotypic means.

**Table 1:** List of the genome-wide significant *cis*-eQTLs detected in the *gluteus medius* (GM) muscle (those that have been consistently detected in the liver are shown in bold)^1^

Muscle *cis-*eQTL										Gene	
SSC	N	SNP	Region (Mb)	*P-*value	*q-value*	*B*	δ ± SE	A_1_	MAF	Acronym	Region (Mb)
1	51	ASGA0000817	0.2–9.9	6.69E-08	2.12E-04	1.91E-03	−0.37 ± 0.06	A	0.388	*ARID1B*	9.7–10.2
1	15	ALGA0002427	32.2–36.6	2.74E-11	5.90E-07	7.80E-07	0.58 ± 0.06	A	0.365	*AKAP7*	32.2–32.3
1	10	INRA0003613	101.9–108.9	3.29E-12	2.00E-08	9.00E-08	−0.29 ± 0.03	C	0.433	*WDR7*	106.0–106.4
1	2	MARC0039560	125.9–128.7	5.67E-06	3.24E-02	1.62E-01	−0.16 ± 0.03	G	0.481	*HYPK*	127.7–127.7
1	5	DIAS0000210	183.3–183.9	5.36E-07	7.63E-03	1.53E-02	0.28 ± 0.05	C	0.466	*CNIH1*	183.8–183.8
1	9	ASGA0006202	231.0–239.7	3.49E-11	8.10E-07	9.90E-07	0.50 ± 0.07	A	0.115	*GRHPR*	238.1–238.1
1	18	M1GA0001375	240.2–249.6	1.92E-13	1.00E-08	1.00E-08	0.27 ± 0.03	A	0.399	*ALG2*	240.9–240.9
1	1	H3GA0004649	260.9–260.9	2.36E-06	3.24E-02	6.73E-02	−0.36 ± 0.07	A	0.17	*PSMD5*	260.8–260.8
**2**	**1**	**ALGA0011482**	**5.8–5.8**	**1.47E-06**	**4.20E-02**	**4.20E-02**	**0.28 ± 0.06**	**A**	**0.144**	***CTSF***	**5.8–5.8**
2	22	MARC0095742	32.5–39.3	4.52E-10	4.30E-06	1.29E-05	−0.53 ± 0.08	A	0.354	*HTATIP2*	39.0–39.0
2	24	ALGA0013197	33.0–39.3	1.24E-14	0.00E+ 00	0.00E+ 00	−0.52 ± 0.06	A	0.25	*PRMT3*	38.8–39.0
2	4	ALGA0115180	115.1–116.1	9.98E-09	2.36E-04	2.84E-04	0.37 ± 0.06	A	0.141	*WDR36*	115.8–115.9
2	8	DIAS0000550	140.2–145.0	1.54E-06	1.97E-02	4.40E-02	−0.31 ± 0.05	G	0.466	*ETF1*	140.4–140.5
2	15	H3GA0055810	147.1–148.7	2.30E-08	6.56E-04	6.56E-04	−0.30 ± 0.05	G	0.16	*LARS*	147.4–147.5
3	1	MARC0045458	3.6–3.6	3.41E-08	9.72E-04	9.72E-04	0.26 ± 0.04	G	0.223	*AP5Z1*	3.6–3.6
3	2	MARC0020406	4.1–4.2	4.17E-08	7.28E-04	1.19E-03	−0.68 ± 0.12	A	0.121	*ZNF12*	4.4–4.4
3	14	ALGA0017913	18.6–19.8	1.33E-09	1.78E-05	3.78E-05	0.30 ± 0.05	G	0.125	*NSMCE1*	19.6–19.6
3	2	DRGA0003838	29.6–29.6	5.05E-07	7.81E-03	1.44E-02	−0.37 ± 0.06	A	0.37	*MKL2*	28.9–29.2
3	26	MARC0101576	30.0–34.4	5.74E-09	1.64E-04	1.64E-04	−0.22 ± 0.04	G	0.345	*RSL1D1*	31.2–31.2
3	17	MARC0000159	48.6–54.6	2.77E-06	9.87E-03	7.89E-02	−0.27 ± 0.06	G	0.194	*MRPS9*	49.6–49.6
3	18	MARC0049447	93.7–97.2	3.90E-07	2.78E-03	1.11E-02	0.29 ± 0.05	A	0.272	*THADA*	96.8–97.1
3	7	M1GA0026608	126.9–127.8	4.55E-09	6.48E-05	1.30E-04	0.58 ± 0.10	A	0.178	*CYS1*	126.3–126.4
4	6	ASGA0018519	14.3–15.8	3.76E-06	3.03E-02	1.07E-01	−0.21 ± 0.04	C	0.393	*NDUFB9*	15.1–15.1
4	2	ASGA0018539	14.6–14.6	9.98E-09	2.84E-04	2.84E-04	0.19 ± 0.03	G	0.322	*WASHC5*	14.5–14.6
4	6	ALGA0024302	30.2–31.5	2.51E-06	9.62E-03	7.15E-02	−0.40 ± 0.07	G	0.433	*OXR1*	30.7–31.2
**4**	**11**	**DIAS0000176**	**95.2–99.8**	**2.07E-07**	**1.97E-03**	**5.91E-03**	**0.33 ± 0.06**	**A**	**0.252**	***APH1A***	**98.8–98.8**
4	4	M1GA0006152	95.3–96.1	3.55E-07	1.01E-02	1.01E-02	−0.23 ± 0.04	G	0.341	*S100A13*	96.0–96.0
5	4	ASGA0104541	20.3–23.5	1.61E-06	1.57E-02	4.59E-02	0.17 ± 0.03	A	0.325	*TSPAN31*	23.0–23.0
5	12	SIRI0000318	25.6–29.4	2.96E-07	2.11E-03	8.43E-03	−0.53 ± 0.10	A	0.306	*TMEM5*	28.2–28.2
5	24	MARC0113779	80.3–84.7	2.66E-10	7.10E-07	7.58E-06	−0.39 ± 0.06	A	0.136	*C12orf73*	80.5–80.5
**6**	**25**	**ASGA0098408**	**17.0–21.7**	**1.11E-08**	**1.40E-04**	**3.16E-04**	**0.49 ± 0.08**	**G**	**0.24**	***ARL2BP***	**19.2–19.2**
6	28	DIAS0004785	45.5–55.4	4.23E-06	1.72E-02	1.21E-01	−0.15 ± 0.03	A	0.471	*RABAC1*	49.9–49.9
**6**	**13**	**ASGA0094600**	**70.1–71.6**	**8.61E-07**	**7.64E-03**	**2.45E-02**	**−0.33 ± 0.06**	**G**	**0.296**	***LZIC***	**70.2–70.2**
6	17	MARC0031169	70.1–71.6	3.65E-06	1.16E-02	1.04E-01	−0.19 ± 0.03	G	0.279	*DFFA*	70.7–70.7
**6**	**36**	**ASGA0028827**	**81.9–89.9**	**5.30E-12**	**1.00E-08**	**1.50E-07**	**−0.39 ± 0.05**	**G**	**0.238**	***BSDC1***	**88.8–88.8**
6	52	MARC0015713	90.6–99.9	2.96E-08	4.21E-04	8.43E-04	−0.20 ± 0.03	A	0.379	*AFG3L2*	97.2–97.2
6	2	ALGA0122704	95.9–99.4	3.18E-06	6.66E-03	9.05E-02	0.37 ± 0.08	A	0.327	*RAB12*	99.2–99.2
6	6	ALGA0037549	157.5–158.8	2.07E-08	5.89E-04	5.89E-04	0.29 ± 0.05	A	0.262	*MRPL37*	158.0–158.1
**6**	**1**	**ASGA0030033**	**158.6–158.6**	**5.27E-06**	**2.15E-02**	**1.50E-01**	**−0.22 ± 0.05**	**G**	**0.359**	***YIPF1***	**158.4–158.4**
7	19	ASGA0035147	86.3–89.1	1.55E-12	4.00E-08	4.00E-08	0.41 ± 0.05	A	0.5	*MTHFD1*	88.4–88.5
7	21	ALGA0043682	92.1–99.8	7.12E-09	2.03E-04	2.03E-04	−0.26 ± 0.04	G	0.333	*DCAF5*	92.7–92.8
**8**	**11**	**ALGA0112294**	**10.6–17.6**	**1.63E-09**	**6.63E-06**	**4.64E-05**	**−0.25 ± 0.04**	**C**	**0.399**	***TAPT1***	**11.3–11.4**
8	6	ALGA0049334	117.4–118.2	9.95E-09	8.85E-05	2.84E-04	−0.29 ± 0.05	A	0.335	*BDH2*	117.9–117.9
9	37	ASGA0042057	20.2–24.2	2.61E-10	7.44E-06	7.44E-06	0.56 ± 0.08	G	0.49	*CTSC*	21.7–21.7
9	9	ASGA0043022	40.3–47.6	8.95E-11	2.55E-06	2.55E-06	−0.28 ± 0.03	A	0.165	*ARHGEF12*	47.4–47.6
**10**	**4**	**ASGA0046443**	**10.0–14.1**	**1.10E-16**	**0.00E+ 00**	**0.00E+ 00**	**−0.92 ± 0.09**	**G**	**0.228**	***MARC2***	**10.1–10.1**
10	11	ASGA0048312	51.7–58.7	5.83E-08	1.66E-03	1.66E-03	−0.33 ± 0.04	A	0.409	*COMMD3*	52.5–52.6
10	2	ALGA0059373	54.1–54.4	4.39E-08	6.26E-04	1.25E-03	0.33 ± 0.06	G	0.298	*PLXDC2*	54.2–54.6
11	3	DIAS0002284	20.7–21.0	3.50E-08	9.98E-04	9.98E-04	−0.21 ± 0.04	A	0.296	*ESD*	20.6–20.6
12	4	ALGA0115312	4.2–4.8	4.24E-07	1.21E-02	1.21E-02	−0.20 ± 0.04	G	0.296	*ST6GALNAC2*	4.9–4.9
12	3	DRGA0011563	4.2–4.8	3.95E-06	3.75E-02	1.13E-01	−0.42 ± 0.07	A	0.17	*EXOC7*	5.3–5.3
12	3	ALGA0064915	10.9–11.6	1.19E-06	1.70E-02	3.39E-02	−0.40 ± 0.08	G	0.291	*ABCA5*	11.0–11.1
**12**	**16**	**MARC0016809**	**20.3–24.8**	**3.26E-12**	**9.00E-08**	**9.00E-08**	**0.49 ± 0.06**	**G**	**0.272**	***PNPO***	**24.1–24.2**
12	2	DIAS0000973	23.5–23.6	1.52E-06	2.34E-02	4.33E-02	0.24 ± 0.04	C	0.257	*MRPL10*	24.0–24.0
12	8	DIAS0000242	40.0–48.8	3.14E-06	2.01E-02	8.96E-02	0.25 ± 0.05	G	0.433	*TMEM98*	42.0–42.1
**12**	**6**	**ASGA0054801**	**44.3–46.5**	**1.28E-06**	**2.18E-02**	**3.66E-02**	**0.20 ± 0.04**	**G**	**0.481**	***TMEM97***	**44.6–44.6**
**12**	**7**	**ALGA0066917**	**50.5–59.3**	**4.57E-11**	**4.50E-07**	**1.30E-06**	**0.37 ± 0.05**	**G**	**0.335**	***RABEP1***	**51.6–51.8**
12	1	ALGA0120796	51.9–51.9	2.27E-07	6.46E-03	6.46E-03	−0.13 ± 0.02	C	0.412	*PSMB6*	52.0–52.0
12	7	MARC0002558	59.0–59.9	3.63E-10	1.03E-05	1.03E-05	0.41 ± 0.05	A	0.35	*ALDH3A2*	59.9–59.9
13	2	ALGA0067450	2.5–2.7	7.69E-07	2.19E-02	2.19E-02	0.34 ± 0.06	A	0.306	*SH3BP5*	2.5–2.5
13	9	ASGA0086643	12.4–14.4	1.64E-12	1.00E-08	5.00E-08	0.52 ± 0.06	A	0.422	*NGLY1*	12.6–12.6
13	5	ALGA0068741	19.4–23.1	1.67E-06	2.38E-02	4.77E-02	−0.16 ± 0.03	G	0.269	*PDCD6IP*	19.5–19.6
13	5	MARC0000523	31.1–32.5	3.32E-11	9.50E-07	9.50E-07	−0.35 ± 0.05	A	0.155	*MAPKAPK3*	33.0–33.1
**14**	**12**	**H3GA0038597**	**6.1–7.2**	**6.65E-13**	**2.00E-08**	**2.00E-08**	**−0.23 ± 0.03**	**A**	**0.383**	***POLR3D***	**6.5–6.5**
14	1	SIRI0001291	22.7–22.7	4.88E-08	1.39E-03	1.39E-03	−0.20 ± 0.03	G	0.291	*PGAM5*	22.7–22.7
14	8	MARC0094155	40.6–43.2	4.74E-10	5.71E-06	1.35E-05	−0.44 ± 0.06	G	0.212	*ACADS*	40.6–40.6
14	5	ASGA0065742	102.7–106.9	3.84E-08	5.48E-04	1.10E-03	−0.34 ± 0.06	A	0.147	*IDE*	103.9–104.1
14	4	MARC0043866	110.5–111.0	2.01E-06	2.86E-02	5.72E-02	0.30 ± 0.05	A	0.49	*COX15*	110.8–110.8
14	11	ALGA0081813	124.1–125.1	1.19E-08	1.70E-04	3.39E-04	0.44 ± 0.07	G	0.277	*CASP7*	124.0–124.0
15	11	ASGA0069526	42.2–49.0	7.12E-13	0.00E+ 00	2.00E-08	1.33 ± 0.16	G	0.067	*SORBS2*	46.5–46.9
15	54	ALGA0085678	54.5–69.7	4.49E-08	1.28E-03	1.28E-03	−0.36 ± 0.06	G	0.112	*UGGT1*	59.1–59.2
15	9	ASGA0070490	116.4–118.5	1.07E-10	3.05E-06	3.05E-06	0.35 ± 0.05	A	0.471	*XRCC5*	118.3–118.4
16	1	ALGA0090252	34.2–34.2	1.21E-06	3.45E-02	3.45E-02	−0.24 ± 0.04	C	0.485	*SLC38A9*	34.7–34.8
16	6	MARC0049616	72.0–73.1	7.13E-08	1.46E-03	2.03E-03	−0.32 ± 0.05	A	0.288	*CCT5*	72.2–72.2
17	2	ALGA0096195	57.0–57.1	9.93E-08	1.42E-03	2.83E-03	−0.36 ± 0.05	A	0.223	*FAM210B*	56.9–56.9
18	5	ALGA0097540	24.4–25.8	1.38E-07	3.94E-03	3.94E-03	0.44 ± 0.08	A	0.142	*FAM3C*	25.5–25.6

^1^SSC: porcine chromosome, N: Number of SNPs significantly associated with the trait under study, SNP: SNP displaying the most significant association with the trait under study, Region (Mb): region containing SNPs significantly associated with the trait under study, *P-*value: nominal *P-*value, *q-value*: *q-value* calculated with a false discovery rate approach, *B*: Bonferroni corrected *P-*values, δ: allelic effect and its standard error (SE), A_1_: minority allele, MAF: frequency of the minority allele

**Table 2:** List of the genome-wide significant *cis*-eQTLs detected in the liver tissue (those that have been consistently detected in the muscle are shown in bold)^1^

liver *cis-*eQTL										Gene	
SSC	N	SNP	Region (Mb)	*P-*value	*q-value*	*B*	δ ± SE	A_1_	MAF	Acronym	Region (Mb)
1	15	H3GA0004318	251.1–254.7	8.56E-10	2.43E-05	2.43E-05	0.49 ± 0.07	A	0.281	*HSDL2*	253.1–253.1
1	1	ASGA0006805	253.4–253.4	9.61E-07	2.73E-02	2.73E-02	0.22 ± 0.04	C	0.411	*CDC26*	253.8–253.9
**2**	**7**	**MARC0045154**	**4.6–8.8**	**2.63E-06**	**3.22E-02**	**7.47E-02**	**0.27 ± 0.06**	**A**	**0.146**	***CTSF***	**5.8–5.8**
2	17	DIAS0003663	82.9–89.7	8.30E-09	1.18E-04	2.36E-04	0.30 ± 0.05	A	0.411	*SERINC5*	88.8–88.9
**4**	**8**	**DIAS0000176**	**96.7–99.8**	**2.13E-07**	**2.27E-03**	**6.04E-03**	**0.30 ± 0.05**	**A**	**0.255**	***APH1A***	**98.8–98.8**
5	1	H3GA0016032	19.8–19.8	8.25E-07	1.17E-02	2.35E-02	−0.38 ± 0.07	G	0.24	*PPP1R1A*	19.7–19.7
**6**	**23**	**ALGA0116876**	**17.0–21.7**	**9.05E-16**	**0.00E+ 00**	**0.00E+ 00**	**0.86 ± 0.08**	**A**	**0.255**	***ARL2BP***	**19.2–19.2**
**6**	**13**	**MARC0031169**	**70.1–71.6**	**9.29E-07**	**3.30E-03**	**2.64E-02**	**−0.34 ± 0.04**	**G**	**0.276**	***LZIC***	**70.2–70.2**
6	1	ASGA0104284	80.6–80.6	1.70E-06	4.82E-02	4.82E-02	−0.20 ± 0.04	C	0.141	*ZNF436*	81.2–81.2
**6**	**14**	**ALGA0114962**	**82.1–89.0**	**6.79E-06**	**1.76E-02**	**1.93E-01**	**−0.32 ± 0.06**	**A**	**0.24**	***BSDC1***	**88.8–88.8**
**6**	**2**	**ASGA0030033**	**158.1–158.6**	**9.06E-07**	**2.57E-02**	**2.57E-02**	**−0.24 ± 0.04**	**G**	**0.328**	***YIPF1***	**158.4–158.4**
7	2	DRGA0007123	9.9–10.4	5.49E-10	1.56E-05	1.56E-05	−0.40 ± 0.05	G	0.24	*NOL7*	9.9–9.9
**8**	**13**	**ALGA0112294**	**10.6–11.3**	**3.37E-08**	**1.37E-04**	**9.58E-04**	**−0.36 ± 0.06**	**C**	**0.406**	***TAPT1***	**11.3–11.4**
9	2	ASGA0094801	46.6–46.7	3.33E-06	4.73E-02	9.46E-02	−0.54 ± 0.10	G	0.266	*THY1*	46.6–46.6
**10**	**1**	**ASGA0046443**	**10.2–10.2**	**4.07E-06**	**1.27E-02**	**1.16E-01**	**−0.25 ± 0.05**	**G**	**0.229**	***MARC2***	**10.1–10.1**
10	10	ALGA0106385	48.1–54.6	1.22E-07	2.04E-03	3.46E-03	0.24 ± 0.04	G	0.396	*KIAA1217*	50.7–51.2
**12**	**2**	**MARC0042683**	**24.1–24.7**	**5.74E-07**	**1.63E-02**	**1.63E-02**	**−0.30 ± 0.06**	**G**	**0.297**	***PNPO***	**24.1–24.2**
12	3	M1GA0016795	42.8–43.1	1.61E-06	2.29E-02	4.58E-02	−0.32 ± 0.06	A	0.464	*CRLF3*	42.9–42.9
**12**	**9**	**ALGA0066653**	**44.0–46.4**	**6.21E-12**	**9.00E-08**	**1.80E-07**	**0.31 ± 0.04**	**G**	**0.5**	***TMEM97***	**44.6–44.6**
**12**	**3**	**ALGA0066917**	**51.0–59.3**	**3.85E-06**	**3.65E-02**	**1.09E-01**	**0.19 ± 0.04**	**G**	**0.333**	***RABEP1***	**51.6–51.8**
13	1	ALGA0070572	61.1–61.1	1.97E-06	2.80E-02	5.60E-02	−0.40 ± 0.07	A	0.474	*ITPR1*	61.0–61.3
13	6	ALGA0103755	141.3–148.9	1.73E-07	1.52E-03	4.92E-03	0.44 ± 0.06	A	0.286	*GTPBP8*	146.9–146.9
**14**	**11**	**H3GA0038597**	**6.5–7.2**	**1.86E-09**	**4.94E-05**	**5.28E-05**	**−0.39 ± 0.06**	**A**	**0.401**	***POLR3D***	**6.5–6.5**
14	22	H3GA0039048	10.1–14.0	8.60E-11	4.10E-07	2.44E-06	0.35 ± 0.05	G	0.469	*HMBOX1*	12.6–12.8
14	2	DRGA0013801	42.7–43.4	2.70E-07	7.66E-03	7.66E-03	−0.33 ± 0.06	A	0.406	*GRK3*	43.3–43.4
14	2	ASGA0065623	100.9–100.9	1.21E-06	1.24E-02	3.43E-02	0.37 ± 0.07	A	0.234	*LIPA*	101.1–101.2
16	5	ALGA0088786	5.7–5.8	1.04E-07	9.88E-04	2.96E-03	0.61 ± 0.10	G	0.406	*RETREG1*	5.7–5.8
18	8	ASGA0100292	0.2–2.3	4.33E-06	3.08E-02	1.23E-01	−0.15 ± 0.03	A	0.234	*ESYT2*	0.5–0.6

^1^SSC: porcine chromosome, N: Number of SNPs significantly associated with the trait under study, SNP: SNP displaying the most significant association with the trait under study, Region (Mb): region containing SNPs significantly associated with the trait under study, *P-*value: nominal *P-*value, *q-value*: *q-value* calculated with a false discovery rate approach, *B*: Bonferroni corrected *P-*values, δ: allelic effect and its standard error (SE), A_1_: minority allele, MAF: frequency of the minority allele

### Co-localization of copy number variants and eQTLs

**Fig. 5:**
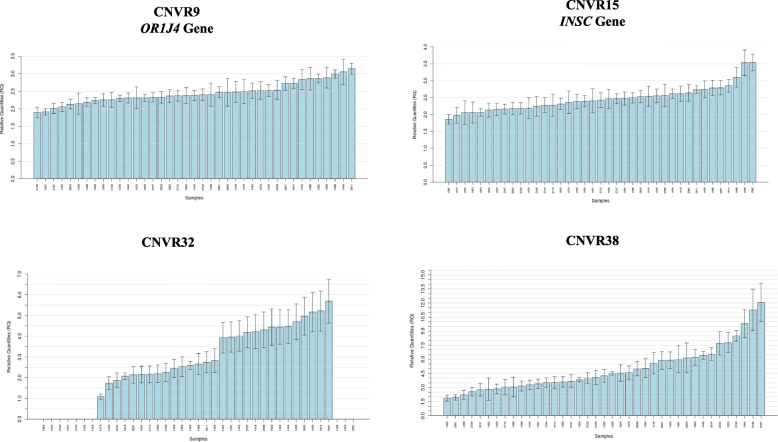
Relative expression values of four copy number variation regions validated by quantitative real-time PCR analysis. Each analysed individual is represented in the x-axis, while the y-axis shows the corresponding relative quantification (RQ) value. We have assigned a value of 2 to the arithmetic mean of the samples used as calibrators

The analysis of structural variation was performed with two softwares: PennCNV and GADA. We detected 93 and 103 CNVRs with PennCNV and GADA, respectively, and 44.08% of the CNVRs detected with PennCNV were also called by GADA. These 41 common CNVRs were distributed along 13 pig chromosomes (Additional file 3). The proportions of copy* gain*, *loss* and *loss/gain* CNVRs were ~ 48.78%, ~ 39.02% and ~ 12.19% respectively. The size of the CNVRs ranged between 31.4 kb and 5.2 Mb, with a mean of 457.4 kb. We compared our CNVR dataset with other CNVRs previously reported in pigs [22–29], and found that 60.97% of our CNVRs had been previously reported (Additional file 4). Real time quantitative assays were designed and used to validate 4 CNVRs (CNVR 9, 15, 32 and 38) in 39 porcine samples. According to D’haene [30], estimates of copy number between 1.414 and 2.449 most likely correspond to a normal copy number of 2, whilst anything below or above these thresholds might represent a deletion (CN = 1) or a duplication (CN = 3), respectively. Following these criteria, the four regions under analysis showed evidence of structural variation (Fig. 5). The co-localization of CNVRs and eQTLs was also analyzed (Additional file 5). In the GM muscle, 2 CNVRs co-localized with 3 *cis*-eQTLs, while 2 CNVRs co-localized with 2 hepatic *cis-*eQTLs. This low concordance between CNVRs and *cis*-eQTLs was not surprising as only 10 CNVRs co-localized with the genomic coordinates of the gene data set analyzed in our experiment.

## Discussion

### The profiles of mRNA expression of the porcine liver and skeletal muscle are highly divergent

Skeletal muscle and liver have been selected as target tissues to perform a comparative eQTL analysis because they have a key role in body energy homeostasis, a parameter that has a strong impact on growth and fat deposition in pigs. The analysis of differential expression revealed that the patterns of expression of these two tissues are highly differentiated in pigs (Fig. 1), probably as a consequence of their distinct embryonic origins and physiological functions [13]. Skeletal muscle and liver also show different patterns of gene expression in mouse [31] and humans [4]. The strongly up-regulated genes in muscle included *BIN1*, which mediates calcium signaling and excitation–contraction coupling [32], tropomyosin (*TPM2*), which is fundamental for muscle contraction [33], and *ZNF106*, which is required for the postnatal maintenance of myofiber innervation by motor neurons [34]. In the liver, we detected a strong overexpression of *SORD*, an enzyme that converts sorbitol into fructose [35], *HSD17B13*, whose inactivation leads to chronic liver disease [36], and *APOA1*, the major structural component of high-density lipoproteins [37].

### Identification of *cis*-eQTLs with consistent effects on the mRNA levels of genes expressed in the liver and skeletal muscle

In pigs, hundreds of eQTLs with effects on the muscle transcriptome have been detected, but the majority of these studies focused on target subsets of genes either mapping to QTLs [12] or displaying significant expression-phenotype correlations [5, 6, 10, 11]. Herewith, we have detected 76 muscle *cis-*eQTLs and 28 liver *cis-*eQTLs which attained genome-wide significance. These numbers are consistent with previous reports. Liaubet et al. [38] made a genome scan based on microarray measurements of *longissimus lumborum* gene expression in 57 pigs and identified 335 eQTLs. Of these, only 18 had *cis*-regulatory effects. Similarly, Cánovas et al. [8] identified 478 skeletal muscle genome-wide significant eQTLs but only 13% acted in *cis-*. Although modest sample size (*N* = 103) limits our ability to identify eQTLs, this sample size ought to allow detecting eQTLs with moderate to large effects on gene expression. As a valid reference, the Genotype-Tissue Expression project detected thousands of eQTLs in 44 tissues characterized by RNA-Seq, and 18 of these tissues were represented by sample sizes equal or below the one used in the current experiment [2]. However, it is also true that sample size largely affects the statistical power of eQTL mapping [4] and thus, the analysis of a larger number of pigs would most likely uncover additional eQTLs that remained undetected in our study.

Our results indicate that almost 42.8% of the *cis-*eQTLs regulating gene expression in the liver also display significant associations with the muscle mRNA levels of the very same genes (Tables 1 and 2, Fig. 2). The liver and muscle common *cis*-eQTLs encompass genes previously related to the function of these two organs, such as the cathepsin F (*CTSF*) gene, that harbors a polymorphism that has been associated with *longissimus dorsi* tenderness, ham weight and fatness in Italian crossbred pigs [39], the transmembrane anterior posterior transformation 1 (*TAPT1*) gene, which was linked to carcass and eviscerated weight in a GWAS in chicken [40], the mitochondrial amidoxime reducing component 2 (*MARC2*) gene, that appears to be involved in porcine fatness [41], and the transmembrane protein 97 (*TMEM97*) gene, which contributes to the regulation of cholesterol levels [42].

There is also a relevant fraction of porcine *cis*-eQTLs that display significant effects only in one of the two tissues (Figs. 3 and 4). In the Genotype-Tissue Expression (GTEx) pilot experiment [4], approximately 50% of eQTLs were shared by the nine human tissues under analysis. Moreover, two main types of eQTLs were especially prevalent, i.e. those that regulate gene expression in a single tissue and those that are ubiquitously detected in all tissues. Interestingly, the GTEx pilot analysis also showed that eQTLs affecting gene expression in the skeletal muscle show a limited replicability in other tissues [4]. In other studies, also performed in humans, the eQTL tissue-specificity ranged between 50% [43] and 60–80% [44], which implies that the effects of many regulatory mutations are modulated by tissue-associated factors.

Several of the *cis-*eQTLs detected in our experiment affected the expression of genes involved in lipid or carbohydrate metabolism. For instance, the down-regulation of *HTATIP2* leads to elevated fatty acid synthesis and enhanced levels of lipogenic enzymes [14]. The *EXOC7 *gene regulates the uptake of fatty acids by adipocytes and *ACADS* is involved in the ß-oxidation of fatty acids [16], while *FAM3C* can suppress hepatic gluconeogenesis [45]. It would be interesting to investigate whether polymorphisms associated with the expression of lipid genes also display associations with fatness traits. Two of the muscle *cis*-eQTLs detected in our study have been previously reported by other authors. An eQTL for the *BSDC1* gene was detected by Ponsuksili et al. [46] and the expression of this gene was also correlated with the percentage of weight loss of the *longissimus dorsi* muscle. Moreover, a local eQTL that regulates the expression of *PNPO* and which co-localizes with several meat quality retail traits (such as the percentage of fat and moisture in meat) was described by Steibel et al. [7]. A remarkable level of heterogeneity has been observed in the genetic determinism of production traits in different porcine breeds [47]. In consequence, we anticipated a limited positional concordance amongst eQTLs detected in different breeds. Indeed, a joint analysis of eQTLs across five human populations revealed that varying linkage disequilibrium patterns across populations results in the detection of large numbers of eQTLs with heterogeneous effects [48].

### Limited positional concordance between *cis*-eQTLs and copy number variation regions

Another goal of our study was to investigate the co-localization of *cis-*eQTLs and CNVRs in order to make an initial assessment of the potential impact of structural variation on gene expression in pigs. With PennCNV and GADA, we consistently detected 41 CNVRs with a mean size of 457.4 kb. When we compared our CNVR dataset with CNVRs previously reported in pigs [22–29], we found that 60.97% of the CNVRs detected by us had been previously reported in the literature (Additional file 4). A moderate agreement of CNVR locations amongst studies and populations has been evidenced in several reports [27, 49–52]. Discrepancies could be due to differences in the genetic background of the populations under analysis, filtering criteria (correction factors, criteria to define CNV and CNVRs, etc.), genotyping methods, CNV calling algorithms and the use of family information [27, 53]. In the GM muscle, 2 CNVRs (14 and 38) co-localized with 2 *cis*-eQTLs (SSC2, 32.6–39.4 Mb and SSC14, 102.7–106.9 Mb), whilst the co-localizations between CNVRs and *cis*-eQTLs in the liver tissue were somewhat similar, i.e. 2 *cis*-eQTLs (SSC2, 5.83–5.90 Mb and SSC13, 148.90–149.17 Mb) co-localized with CNVR 11 and 36. This limited positional concordance is probably due to the fact that only 10 CNVRs co-localized with the set of probes analyzed in the current experiment. The low positional concordance between CNVRs and eQTLs could be also due to technical reasons related to the difficulties of detecting CNVs with SNP arrays and the limited sensitivity and poor annotation of porcine microarrays. Besides, the search of CNVs was performed in a population of 350 individuals, while only 103 pigs had available gene-expression data. On the other hand, variations in copy number do not necessarily involve changes in gene expression, e.g. in heterozygous individuals, the loss of one allele can be compensated by an increase in the expression of the other allele, and duplication can generate additional copies which expression is silenced [53]. A future objective would be to investigate the association between the number of copies of genes and their expression levels, but a high throughput method allowing the accurate determination of CNV genotypes will be needed to achieve this goal (genotype determination based on 60 K SNP array data is too imprecise to carry out such analyses).

## Conclusions

Despite the fact that there are considerable differences in the gene expression patterns of the porcine liver and skeletal muscle, we have identified a substantial proportion of common *cis-*eQTLs regulating gene expression in both tissues.

## Materials and methods

### Phenotyping and genotyping of a commercial Duroc population

We have used a commercial Duroc line of 350 Duroc barrows that were slaughtered at the age of ~ 190 days, with an approximate live weight of 122 kg. This population was generated by crossing 5 boars with ~ 400 sows and selecting one offspring/sow (50 piglets did not complete their productive cycle so we ended up with 350 barrows with valid records). The 350 barrows were bred in the experimental farm of the Pig Control Center of the Institut de Recerca i Tecnologia Agroalimentàries (IRTA). The specific conditions of management and feeding have been previously reported [54, 55]. Pigs were slaughtered in a commercial abattoir following the guidelines of the Spanish Royal Decree 54/1995 (January 20, 1995) to preserve animal welfare. In this way, swine were stunned with carbon dioxide (70% or higher) until they lost consciousness and they were subsequently bled by making an incision in, at least, one carotid artery. At slaughter, GM muscle and liver biopsies were obtained for 103 pigs. Total RNA purification procedures have been previously reported [8, 56]. All experimental procedures were approved by the Ethical Committee of IRTA.

Genomic DNA was extracted from blood samples by following a standard phenol-chloroform protocol. Each pig was genotyped for 62,163 SNPs with the Porcine SNP60 BeadChip (Illumina, SanDiego, CA). The quality of the genotyping results was evaluated with the GenomeStudio software (Illumina). The PLINK software [57] was used to filter out the SNP markers with minor allele frequency below 5%, missing genotype rate above 10%, and displaying significant departures (*P-*value < 0.001) from the Hardy-Weinberg equilibrium. Markers which could not be mapped to the Sscrofa11.1 assembly or that mapped to either the X or Y chromosomes were also eliminated from the data set. Markers in complete linkage disequilibrium (r^2^ > 0.98) were also purged. The final data set used for performing eQTL analyses contained 28,493 SNPs. The filtering criteria used for the CNV analysis was different and only SNPs that did not map to the Sscrofa11.1 assembly or that were located in sex chromosomes were removed, leading to a final number of 46,537 used SNPs.

### Differential expression analysis between muscle and liver tissue

*Gluteus medius* muscle and liver samples were collected from 103 Duroc pigs (Lipgen population) after slaughtering, and immediately frozen in liquid nitrogen. These 103 pigs were selected on the basis of a principal component analysis focused on 13 lipid and growth related traits [58]. We chose individuals representing two different metabolic types, i.e. (i) fat pigs with high intramuscular fat (high saturated and monounsaturated fatty acid content) and also high serum lipid levels, and (ii) pigs that were lean and displayed a low level of intramuscular fat (high polyunsaturated fatty acid content) and circulating lipids [58]. Total RNA was extracted from both GM and liver samples, and mRNA expression profiles were characterized by hybridization to the GeneChip Porcine arrays (Affymetrix Inc., Santa Clara, CA), as previously reported by Cánovas et al. [58]. Hepatic and muscular microarray expression data were deposited in the Gene Expression Omnibus (GEO) public repository, and are accessible through GEO Series accession number GSE115484. The Robust Multi-array Average (RMA) algorithm [59] was employed for carrying out data pre-processing, background correction, normalization and log-transformation of expression values. Gene Intensity level of significance for detecting expressed probes was calculated with the MAS 5.0 algorithm [60]. Control probes and those probes that did not show expression levels above the detection threshold in all samples were filtered out. Differential expression analysis between the GM muscle and liver followed the guidelines of the limma-trend pipeline [61, 62]. The limma’s empirical Bayes method incorporated a mean-variance trend, thus making possible to adequately model the relationship between variance and gene signal intensity. In our study, fold-change (FC) values reflect the mean expression levels in the GM muscle vs liver. The correspondence between differentially expressed probes (|FC| > 1.5; *q-value* < 0.05) and genes was based on the Affymetrix porcine annotation data assembled database [63] and the Biomart database (https://www.ensembl.org/biomart/martview).

### Genome scan for expression QTLs

The genome scan for eQTLs targeted a total of 3326 probes that are simultaneously expressed in both tissues. These genes were selected using the following criteria: The MAS 5.0 [60] algorithm was used to detect the probes with expression levels above the Gene Intensity level of significance. Probes were retained when expressed in all the samples analyzed in both tissues. Expressed probes where then annotated according to Biomart database available at Ensembl repositories (https://www.ensembl.org/biomart/martview) and those with no gene correspondence were removed from further analyses. We used the GEMMA software [64] and followed the methods described by González-Prendes et al. [56] to carry out the association analyses. The fixed effects and parameters assumed in the statistical model were:

$$ y=\mathrm{W}\upalpha +\mathrm{x}\beta +\mathrm{u}+\upvarepsilon $$

where ***y*** is the vector that defines the expression of each gene in the GM muscle and liver of the *i*^*th*^ individual; **W** is the matrix with a column of 1 s and the fixed effects, i.e.“batch of fattening” (with 4 categories) and “laboratory” (microarray data were generated in two different laboratories); **α** is a c-vector of the corresponding coefficients including the intercept; **x** is an n-vector of marker genotypes; ***β*** is the SNP allelic effect estimated as a regression coefficient on the corresponding x genotype (values − 1, 0, 1); **u** is a n-vector of random effects with a n-dimensional multivariate normal distribution MVN_n_ (0, λ *τ* ^− 1^ K) where *τ*^− 1^ is the variance of the residual errors; **λ** is the ratio between the two variance components; **K** is a known relatedness matrix derived from SNPs and **ε** is the vector of errors with an MVN_n_ (0, *τ* ^− 1^ I _n_) being I_n_ the identity matrix. The false discovery rate approach reported by Benjamini and Hochberg [65] was used to correct for multiple testing at the genome-wide level. In this study, *cis-*eQTLs were defined as these genomic regions with SNPs significantly associated with a probe located at a maximum distance of ±1 Mb of the associated SNPs. Co-localization was defined as an overlap of at least 1 base pair between the genomic locations of two eQTLs. The GM muscle *cis*-eQTLs detected with microarrays were validated by considering an RNA-seq data set corresponding to the same tissue and comprising 52 Duroc pigs [66]. These 52 pigs were a subset of the 103 pigs investigated in the current work and the methods to generate GM muscle RNA-seq data are fully described in [66].

### Detection of copy number variation

The PennCNV software [67] was employed to detect copy number variants (CNVs) on the basis of the information provided by 46,537 autosomal SNPs. The PennCNV software implements a hidden Markov model (HMM) to infer CNV calls for each genotyped sample using as input the intensity signal Log R Ratio (LRR) and the B Allele Frequency (BAF) information generated with the BeadStudio software (Illumina). Samples with a standard deviation of LRR > 0.30 and BAF drift > 0.01 were discarded. Besides, a wave adjustment procedure for genomic waves was carried out [67]. With these filtering steps, 20 samples were eliminated from the data set. Only CNVs spanning three or more consecutive SNPs were taken into account. The CNV calling was performed using the default parameters of the HMM model by assuming a UF factor of 0.01. Copy number variant regions (CNVRs) were created by merging CNVs with an overlap of 80% or more. Additionally we used the Genome Alteration Detection Analysis (GADA) package [68] to further validate the CNVR detected with PennCNV and minimize the rate of false positives. The GADA software uses a sparse Bayesian algorithm, based on LRR and BAF values obtained along the genome with the BeadStudio software (Illumina), to identify genomic locations where copy number changes occur. Briefly, a LRR average of markers in a chromosome segment is computed and compared to the median of the respective chromosome. Finally, the copy number class is classified as *gain, loss* or *gain/loss*. In our study, copy number variations spanning three or more consecutive SNPs were taken into account and the multiple array analysis option was employed. The parameters defined for the Bayesian learning model and the backward elimination were: 0.8 for the sparseness hyperparameter and 8 for the critical value of the backward elimination.

### Validation of copy number variant regions by quantitative PCR

Quantitative real time PCR (qPCR) assays were used to validate four CNVRs in 39 porcine samples randomly selected from the Lipgen population. The relative quantification (RQ) of the CNVRs was done as previously described [24]. Primers (Additional file 6) were designed with the Primer Express Software (Applied Biosystems). Copy number variant regions were quantified in 384-well plates using SYBR Select Master Mix in a QuantStudio 12 K Flex Real-Time PCR System platform (Applied Biosystems, Inc., Foster City, CA). Reactions were performed in triplicate, and they contained 7.5 ng of genomic DNA and primers at 300 nM in a final volume of 15 μl. The thermocycling profile was: one cycle at 95 °C for 10 min plus 40 cycles of 15 s at 95 °C and 1 min at 60 °C. Moreover, a melting curve profile (95 °C for 15 s, 60 °C for 15 s and a gradual increase in temperature with a ramp rate of 1% up to 95 °C) was implemented to maximize the specificity of the amplification reactions. Relative expression values were calculated with the Qbase+ software (Biogazelle, Ghent, Belgium) by applying the 2^-ΔΔCt^ method (after verifying that its assumptions were adequately fulfilled) [69]. Relative expression values were calibrated using the arithmetic mean of 3–5 samples showing the lowest number of copies for each specific assay. In the specific case of CNVR32, which encompasses a deletion, the five samples chosen for calibration were those with RQ values around 2. Normalization of the expression data was done by using a previously reported assay based on the glucagon gene [70].

## Additional files

Additional file 1: **Table S1.** List of genes that are differentially expressed (DE) in the *gluteus medius* muscle and the liver (*q*-value < 0.05). (XLSX 353 kb)

Additional file 2: **Figure S1.** Boxplots depicting the mRNA expression levels of *cis*-eQTL regulated genes measured with RNA-Seq and microarrays in the *gluteus medius* muscle of 52 and 103 Duroc pigs, respectively. Means were compared with a Student’s t- test: *P*-value > 0.05 (ns); *P*-value ≤ 0.05 (*); *P*-value ≤ 0.01 (**); *P*-value ≤ 0.001 (***) and *P*-value ≤ 0.0001 (****). (DOCX 807 kb)

Additional file 3: **Table S2.** Locations, absolute frequencies, lengths and status of CNVRs detected in 350 Duroc pigs with the PennCNV and GADA softwares. (XLSX 12 kb)

Additional file 4. **Table S3**. Positional coincidence of the copy number variant regions (CNVR) detected in the current work with CNVRs reported in other pig populations. (XLSX 10 kb)

Additional file 5: **Table S4.** Co-localization of CNVRs and *cis*-eQTLs in the *gluteus medius* muscle (eQTL and gene positions are expressed in Mb). **Table S5.** Co-localization of CNVRs and *cis*-eQTLs in liver tissue (eQTL and gene positions are expressed in Mb). (XLSX 13 kb)

Additional file 6: **Table S6.** Sequences of the primers used to validate CNVRs by qPCR. (XLSX 9 kb)

## Data Availability

Microarray expression data can be found in 10.6084/m9.figshare.7952054, while Porcine SNP60 BeadChip data can be accessed at 10.6084/m9.figshare.7473092.v2

## Abbreviations

|FC|: absolute value of the fold-change
bp: base pair
CNV: Copy number variant
CNVR: Copy number variant regions
DE: Differentially expressed
e.g.: for example
eQTL: expression quantitative trait loci
GEMMA: Genome-wide Efficient Mixed-Model Association
GEO: Gene Expression Omnibus
GM: *gluteus medius*
GWAS: genome-wide association analysis
i.e.: in other words
Kb: Kilobase pairs
Mb: Megabase pairs
mRNA: messenger RNA
SNP: Single nucleotide polymorphism
SSC: porcine chromosome
