# Supplementary material for: About the existence of common determinants of gene expression in the porcine liver and skeletal muscle
Source: BMC Genomics. 2019 Jun 24;20:518. doi: 10.1186/s12864-019-5889-5 (PMC6591854; doi:10.1186/s12864-019-5889-5)

Figure S1. Boxplots depicting the mRNA expression levels of 50 *cis*-eQTL regulated genes measured with RNA-Seq and microarrays in the gluteus medius muscle of 52 and 103 Duroc pigs, respectively. Means were compared with a Student’s t- test: *P*-value > 0.05 (ns); *P-*value ≤ 0.05 (*); *P*-value ≤ 0.01 (**); *P*-value ≤ 0.001 (***) and *P*-value ≤ 0.0001 (****).


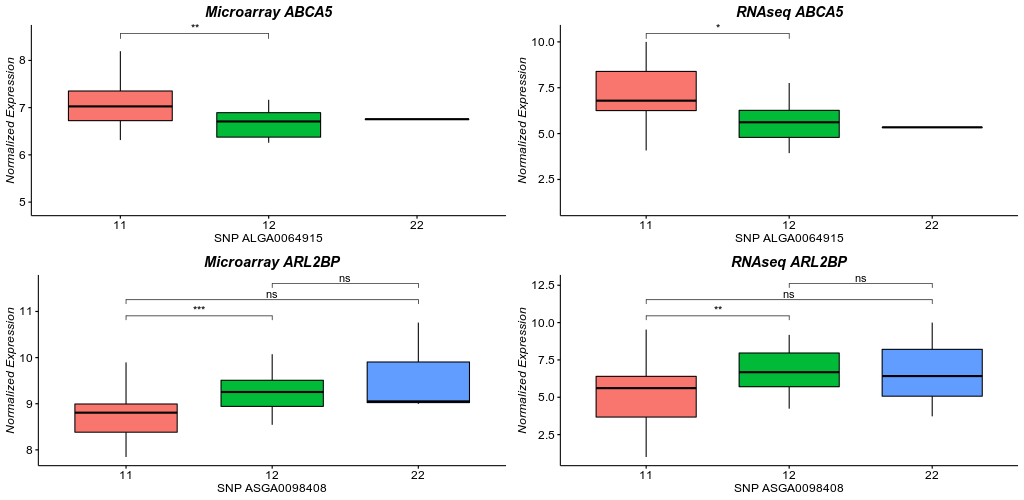

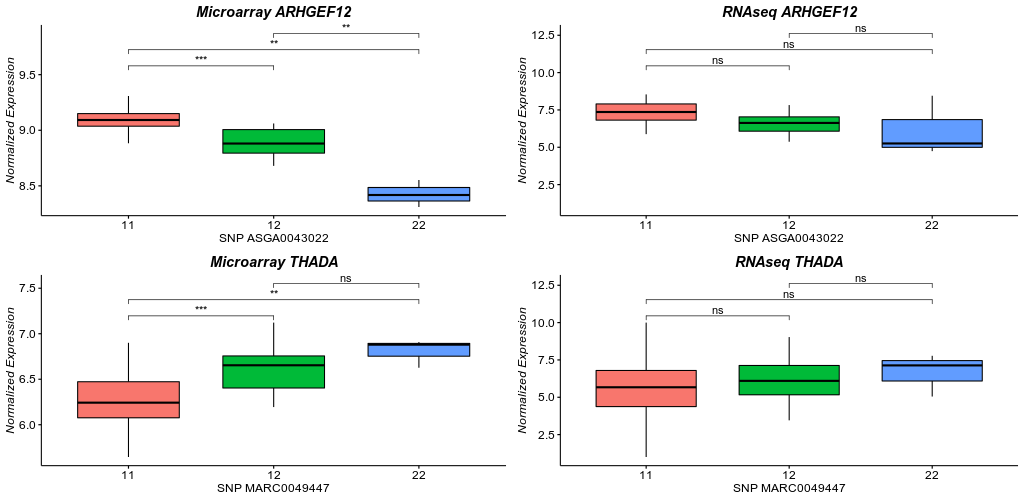

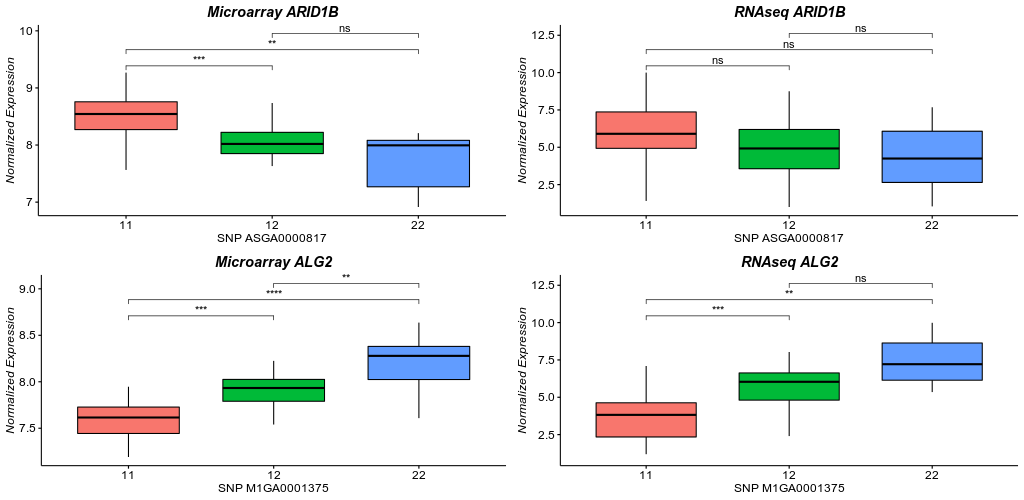

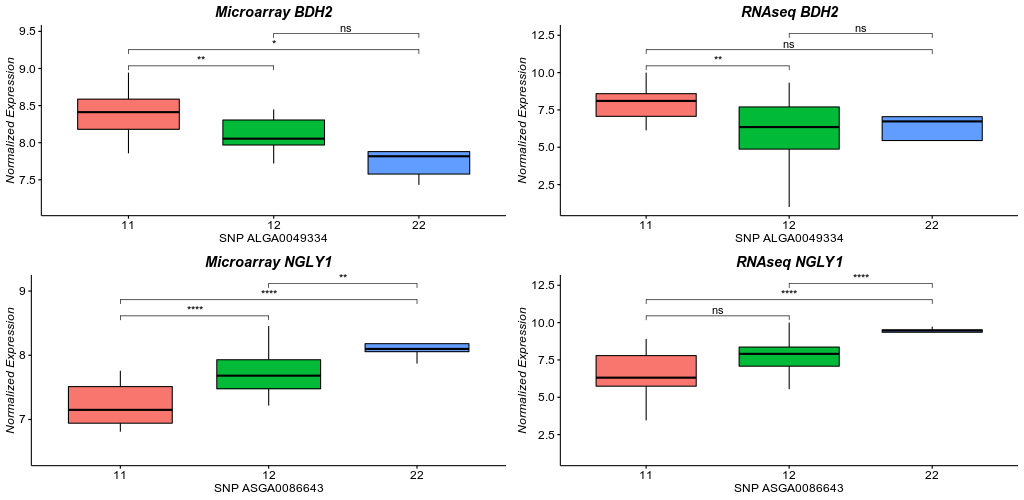

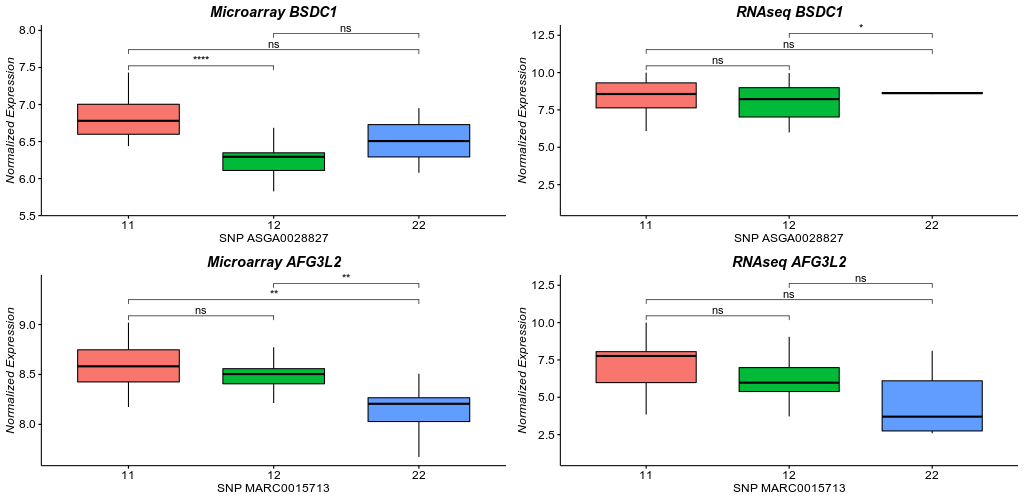

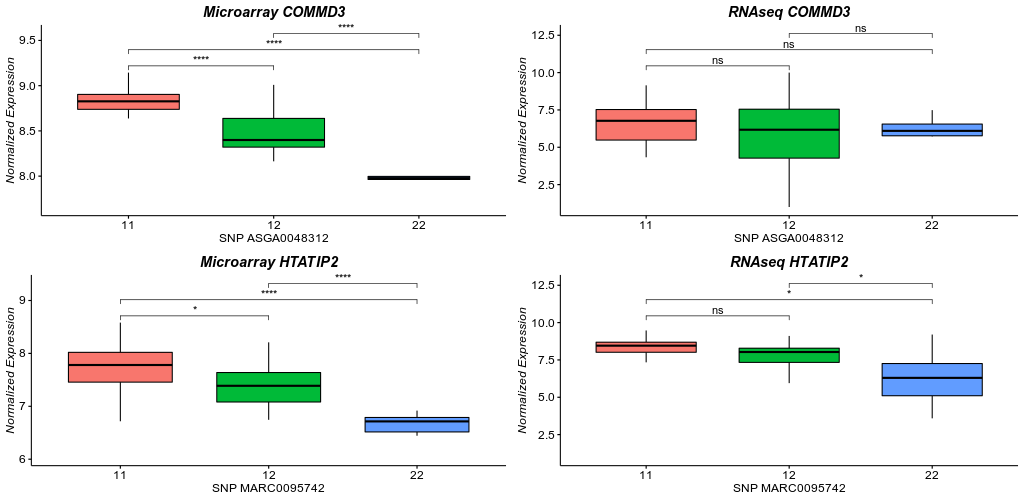

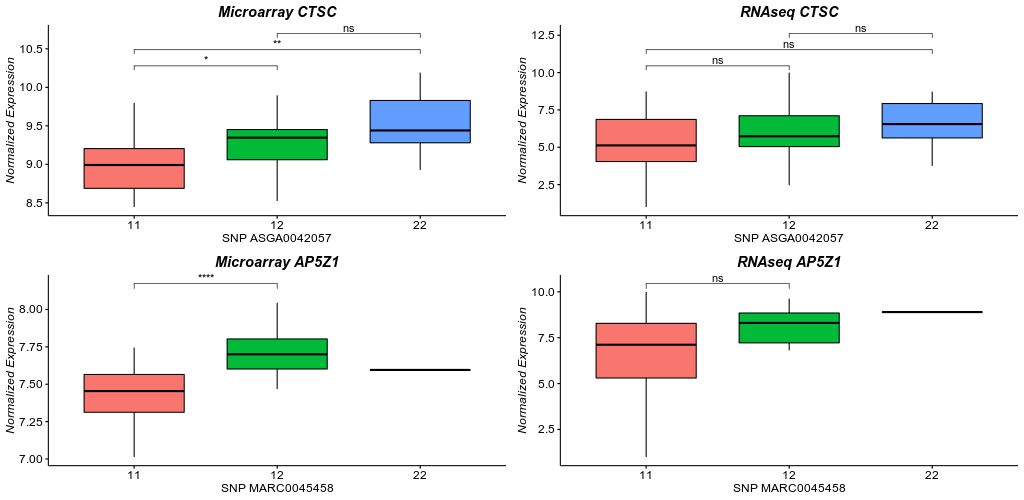

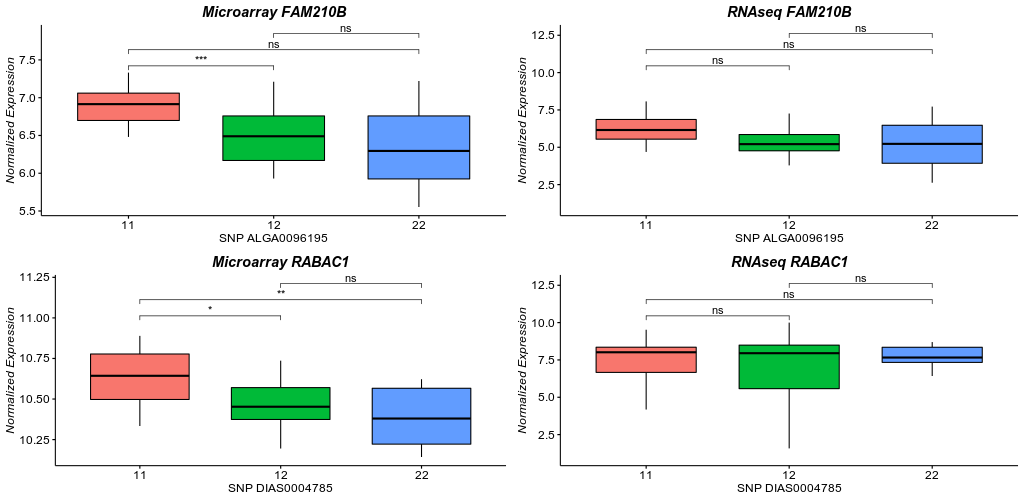

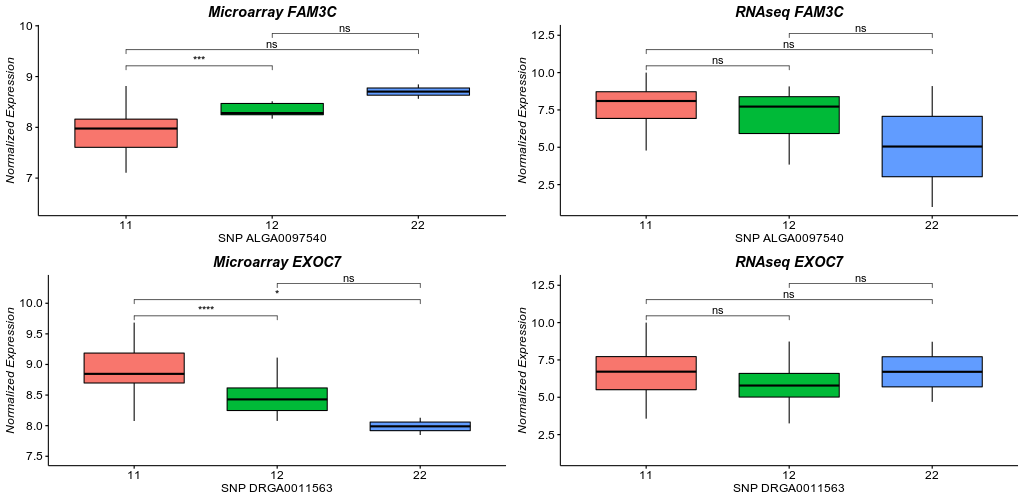

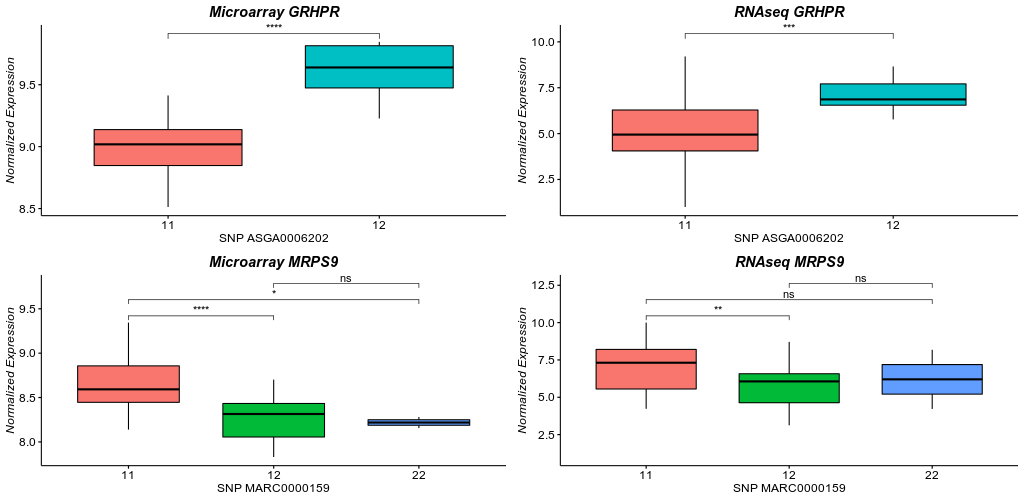

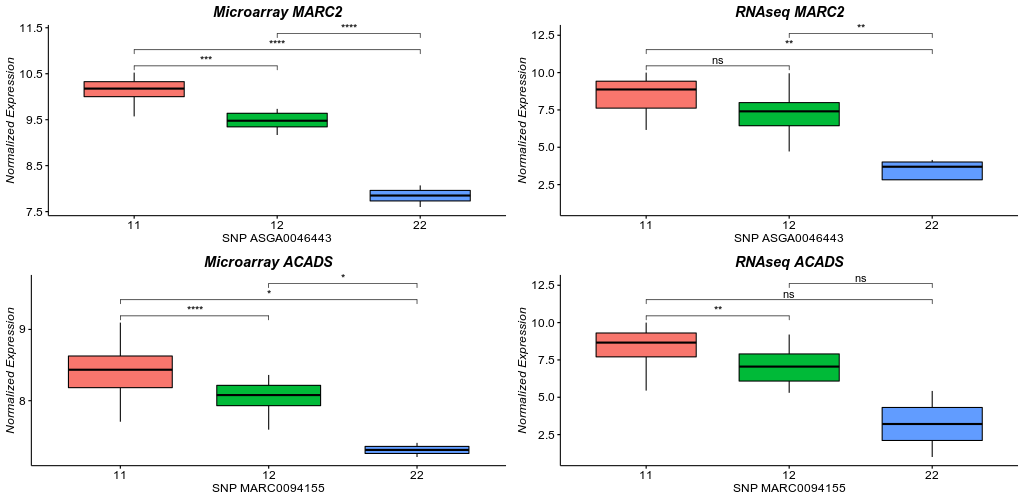

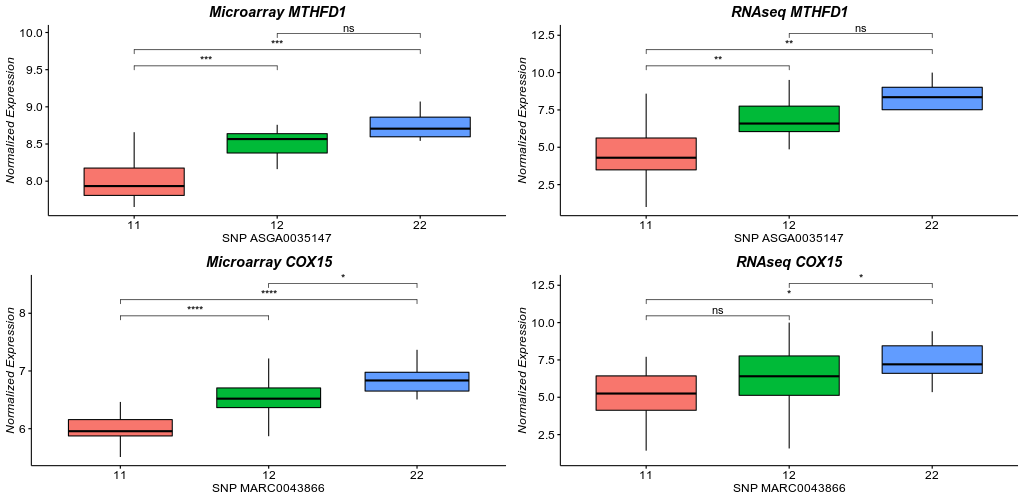

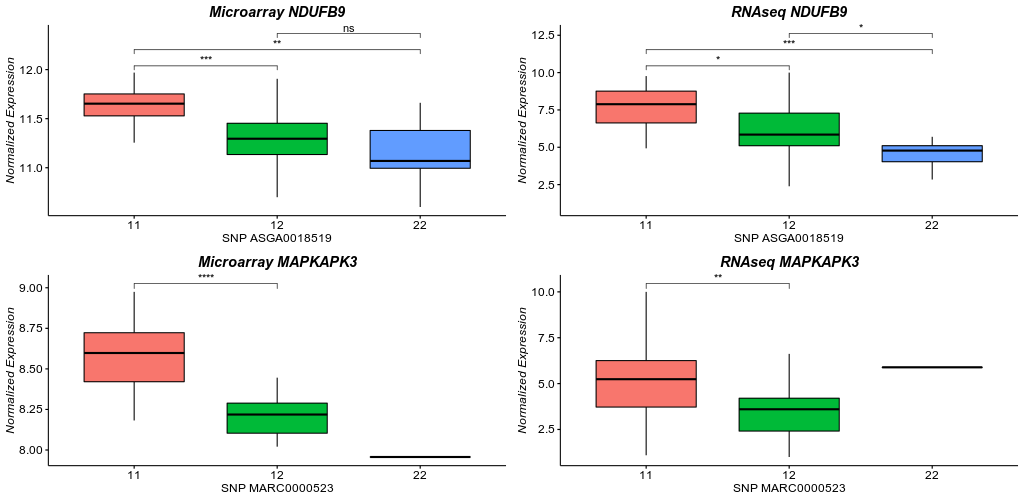

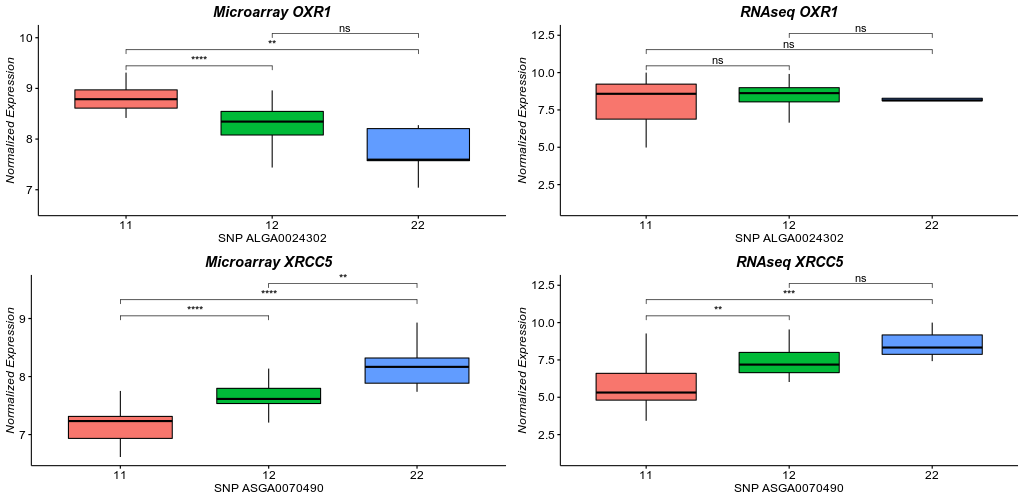

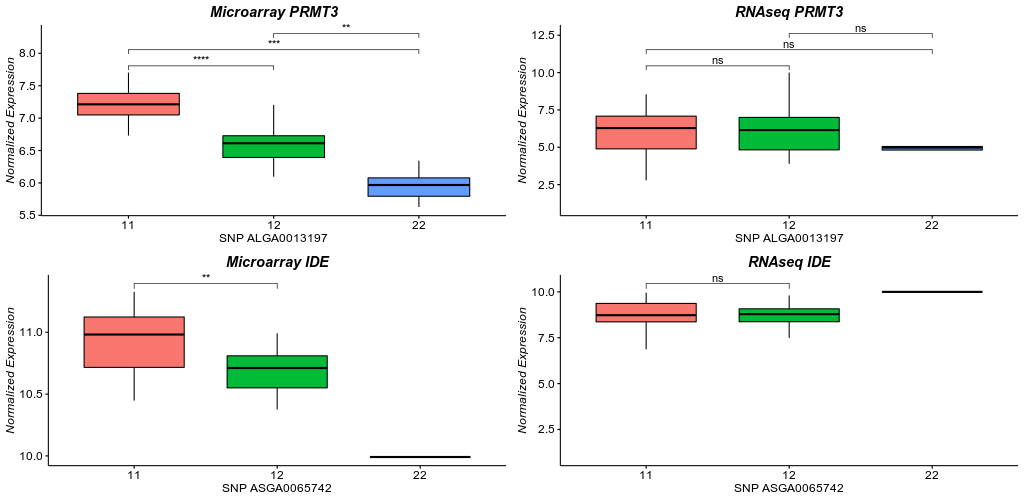

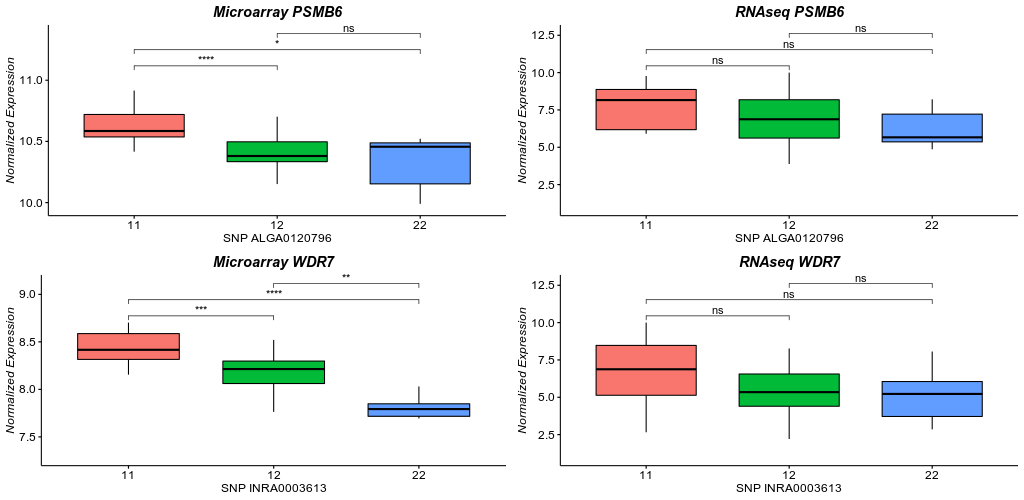

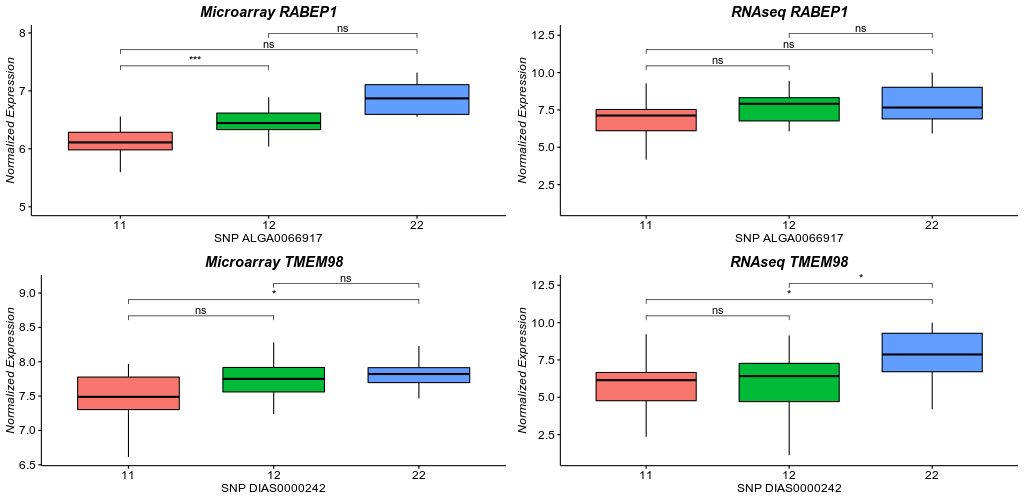

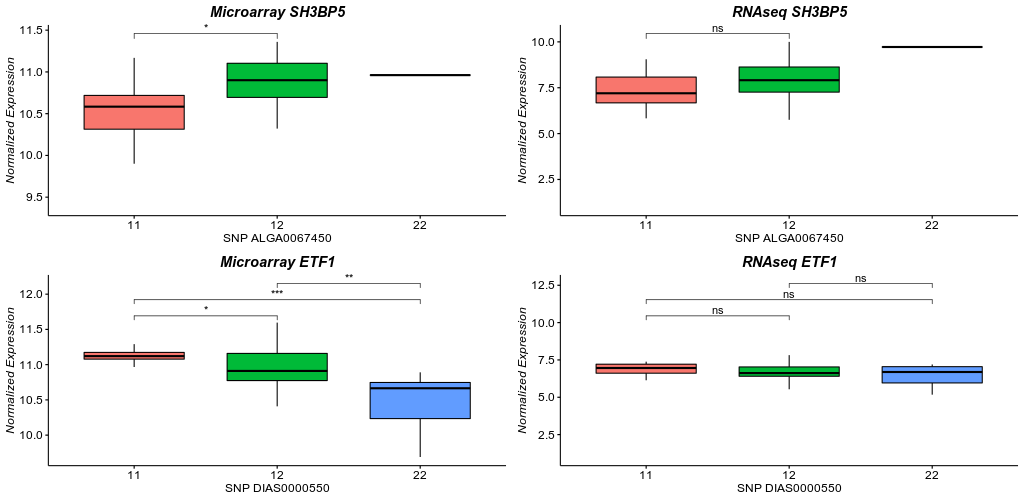

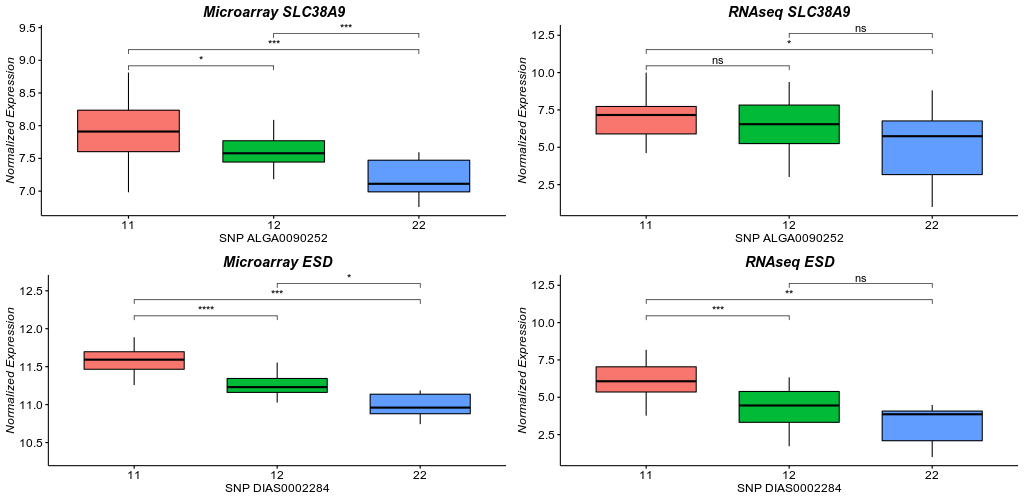

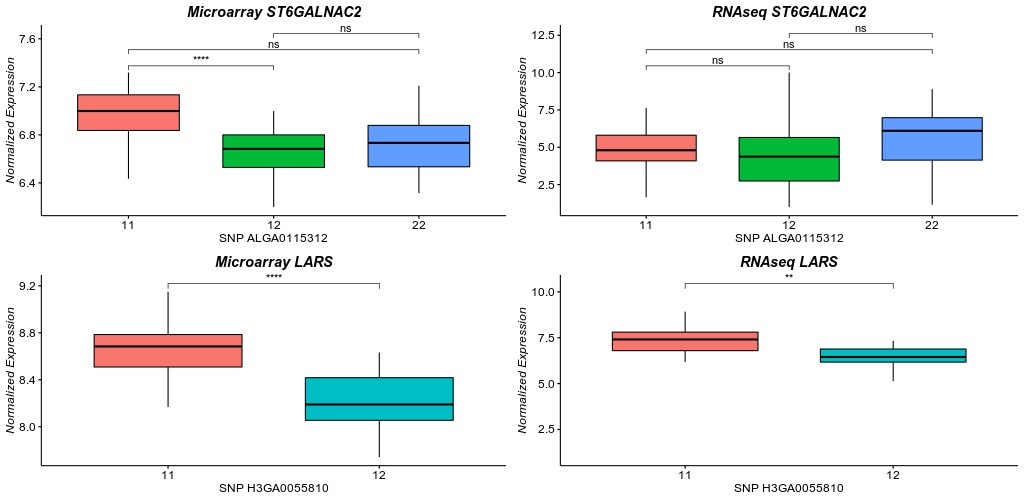

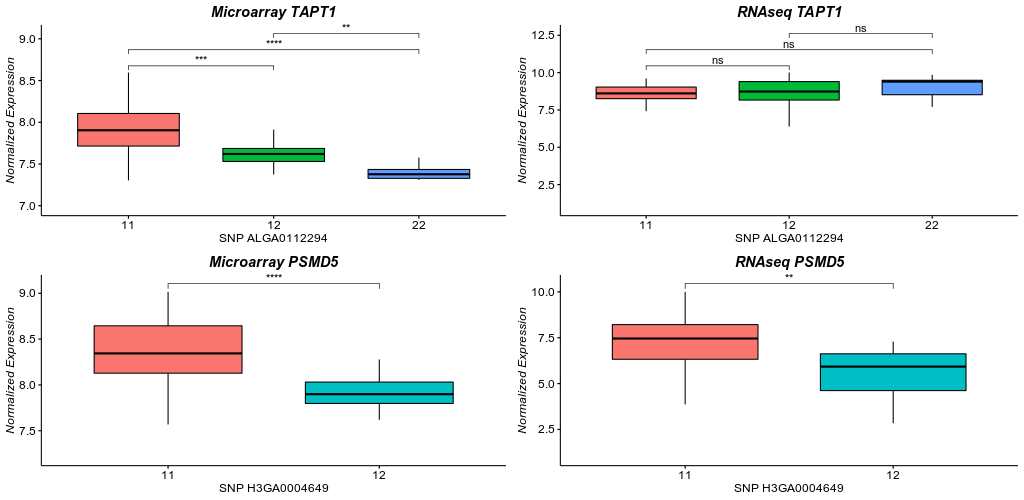

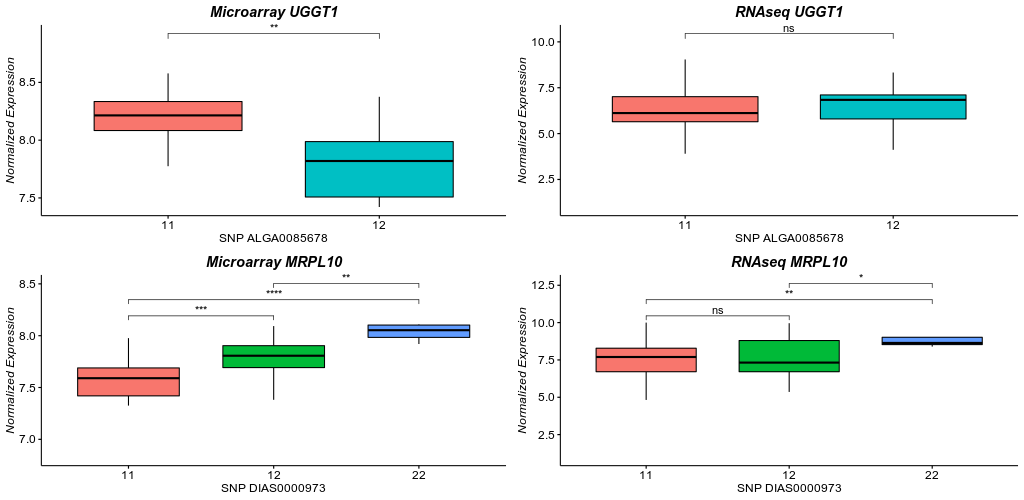

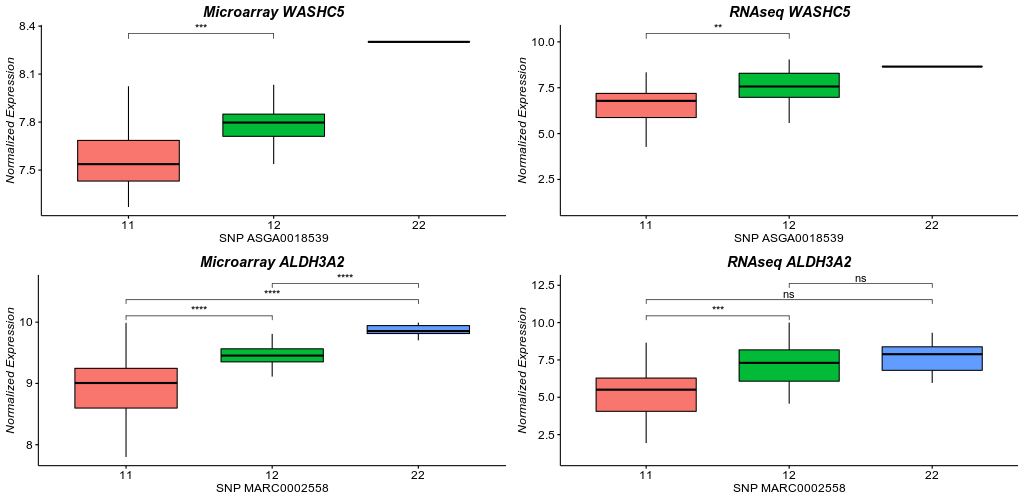

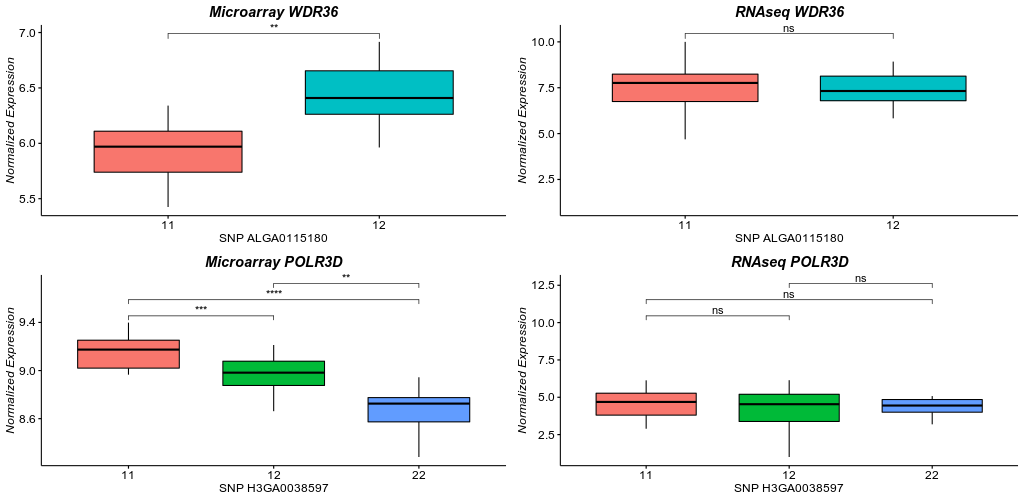

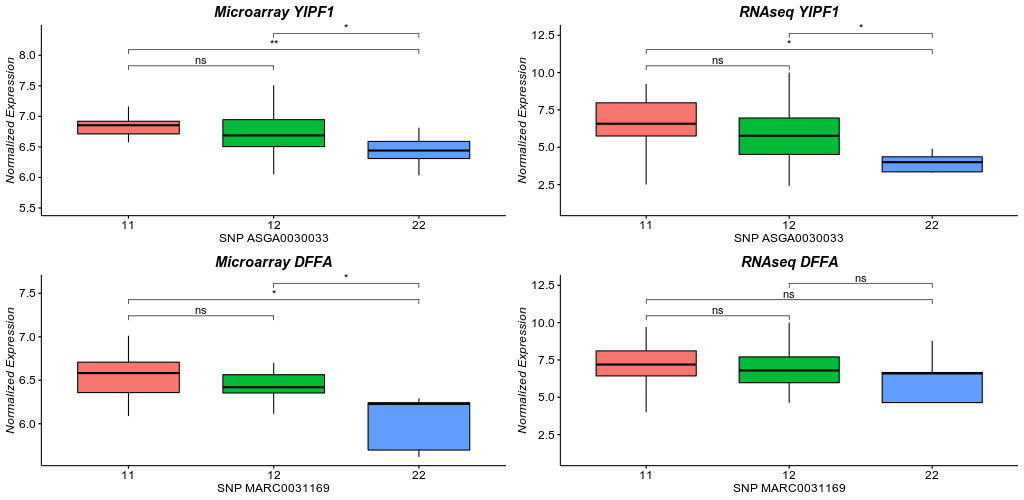

Supplement: Supplementary file 2 — Figure S1. Boxplots depicting the mRNA expression levels of cis-eQTL regulated genes measured with RNA-Seq and microarrays in the gluteus medius muscle of 52 and 103 Duroc pigs, respectively. Means were compared with a Student’s t- test: P-value > 0.05 (ns); P-value ≤ 0.05 (*); P-value ≤ 0.01 (**); P-value ≤ 0.001 (***) and P-value ≤ 0.0001 (****). (DOCX 807 kb) [file 12864_2019_5889_MOESM2_ESM.docx]
